# Mechanical Structure Design and Motion Simulation Analysis of a Lower Limb Exoskeleton Rehabilitation Robot Based on Human–Machine Integration

**DOI:** 10.3390/s25051611

**Published:** 2025-03-06

**Authors:** Chenglong Zhao, Zhen Liu, Yuefa Ou, Liucun Zhu

**Affiliations:** 1 Guangxi Key Laboratory of Ocean Engineering Equipment and Technology, Beibu Gulf University, Qinzhou 535011, China; zhaochenglong@bbgu.edu.cn (C.Z.); ouyuefa@bbgu.edu.cn (Y.O.); 2 Department of Integrated Systems Engineering, Nagasaki Institute of Applied Science, Nagasaki 851-0193, Japan; 3 Advanced Science and Technology Research Institute, Beibu Gulf University, Qinzhou 535001, China; 4 School of Rehabilitation Medieine, Wenzhou Medical University, Wenzhou 325035, China; 5 School of Computer, Shandong Xiehe University, Jinan 250109, China; 6 Institute of Informatics, Kanagawa University, Yokohama 221-8686, Japan

**Keywords:** lower limb exoskeleton, rehabilitation robot, structural design, kinematic simulation

## Abstract

Population aging is an inevitable trend in contemporary society, and the application of technologies such as human–machine interaction, assistive healthcare, and robotics in daily service sectors continues to increase. The lower limb exoskeleton rehabilitation robot has great potential in areas such as enhancing human physical functions, rehabilitation training, and assisting the elderly and disabled. This paper integrates the structural characteristics of the human lower limb, motion mechanics, and gait features to design a biomimetic exoskeleton structure and proposes a human–machine integrated lower limb exoskeleton rehabilitation robot. Human gait data are collected using the Optitrack optical 3D motion capture system. SolidWorks 3D modeling software Version 2021 is used to create a virtual prototype of the exoskeleton, and kinematic analysis is performed using the standard Denavit–Hartenberg (D-H) parameter method. Kinematic simulations are carried out using the Matlab Robotic Toolbox Version R2018a with the derived D-H parameters. A physical prototype was fabricated and tested to verify the validity of the structural design and gait parameters. A controller based on BP fuzzy neural network PID control is designed to ensure the stability of human walking. By comparing two sets of simulation results, it is shown that the BP fuzzy neural network PID control outperforms the other two control methods in terms of overshoot and settling time. The specific conclusions are as follows: after multiple walking gait tests, the robot’s walking process proved to be relatively safe and stable; when using BP fuzzy neural network PID control, there is no significant oscillation, with an overshoot of 5.5% and a settling time of 0.49 s, but the speed was slow, with a walking speed of approximately 0.18 m/s, a stride length of about 32 cm, and a gait cycle duration of approximately 1.8 s. The model proposed in this paper can effectively assist patients in recovering their ability to walk. However, the lower limb exoskeleton rehabilitation robot still faces challenges, such as a slow speed, large size, and heavy weight, which need to be optimized and improved in future research.

## 1. Introduction

With the global population aging rapidly, the World Health Organization (WHO) estimates that, by 2050, the number of people over 60 years old will reach 2 billion, and those over 65 will account for 15.6% of the population [1]. Among them, the number of individuals suffering from hemiplegia, paraplegia, and elderly people with walking difficulties is increasing significantly. The WHO reports that approximately 15 million people worldwide suffer from a stroke each year [2], and between 250,000 and 500,000 people experience spinal cord injuries [3]. These trends pose severe challenges to social development and aging care, creating a growing demand for assistive robots.

To address these challenges, lower limb exoskeleton rehabilitation robots have emerged as a promising solution. These robots can assist in rehabilitation training and offer personalized gait plans tailored to individual patients. AmirHossein MajidiRad et al. experimentally verified the effect of a lower limb exoskeleton on knee rehabilitation efficacy [4]. Recent advances in biosignal interfaces, particularly electromyography (EMG), have significantly enhanced the control and functionality of these systems. Research has shown that incorporating EMG signals enables more intuitive human–machine interaction, with recent studies demonstrating improved gait phase recognition through phasor-based myoelectric features [5] and precise ankle position control using neuromechanical-driven approaches [6]. These developments highlight the importance of integrating biosignal processing with mechanical design to achieve more natural and effective rehabilitation outcomes.

The initial research focus on exoskeleton robots primarily aimed at enhancing the wearer’s ability to carry heavy loads. However, with advances in medical theories and robotics, the focus has shifted toward rehabilitation. Notable examples include the LOKOMAT developed by HOCOMA and the Balgrist Rehabilitation Medical Center, which allows patients to perform active training on a treadmill [7]. Visharath Adhikari et al. designed and implemented a novel task-based on-demand assistive controller and patient rehabilitation tracking system for knee rehabilitation exoskeleton devices [8]. Xin Jin et al. proposed an ESO-based predictive control strategy for lower limb rehabilitation exoskeleton interference suppression model, which helps patients perform better rehabilitation training [9]. Lu Yi’s team proposed a novel 6-DoF 3UPS parallel manipulator. Through kinematic and static modeling, they derived analytical formulas for displacement, velocity, acceleration, and driving forces and analyzed the collaborative motion characteristics between the manipulator and its multi-finger system. Simulations validated the accuracy of the theoretical model and demonstrated that its workspace outperforms the traditional 6/6SPS Stewart platform [10]. Additionally, the team developed a rigid–flexible–soft hybrid finger mechanism integrated with a standard force sensor, combining rigid actuation and flexible deformation capabilities. By constructing an equivalent mechanism model, they derived kinematic and static equations for fingertip motion and analyzed the influence of spring deformation on gripping forces. Experiments confirmed that the finger achieves large-range bending, adaptive contact, and precise force control, effectively reducing impact and ensuring secure grasping [11], and the NaTUre.gaits robot developed by Nanyang Technological University supports ground walking rehabilitation with a weight reduction system [12]. Additionally, the HAL series exoskeletons developed by Cybernics Lab at the University of Tsukuba were the first commercially available products to assist elderly people and those with lower limb disabilities in walking [13]. Other developments include the BLEEX lower-limb exoskeleton developed by the University of California, Berkeley, which uses bionic mechanical legs to assist in walking [14].

Despite these advancements, traditional rigid robot structures and position control methods often introduce safety risks, limiting their widespread use. To address this, soft robots have become a key area of research. For example, FANUC’s CR-35IA collaborative robot automatically detects contact with humans and stops when a dangerous situation occurs [15]. Similarly, the StarIETH robot developed by ETH Zurich uses a flexible structure to reduce impact and energy consumption [16].

This paper proposes a human–machine integrated lower limb exoskeleton rehabilitation robot, combining biomechanical principles with cutting-edge robotics technology. Human motion data are first collected using the Optitrack optical 3D motion capture system, which is then used to derive joint angle changes that inform the design. A virtual prototype of the robot is created using SolidWorks, and kinematic simulation analysis is conducted with the Matlab Robotic Toolbox. Finally, a physical prototype is built to verify the structural design and gait parameters. The contributions of this work include the integration of human biomechanics into robot design and the development of a practical prototype that can assist with rehabilitation training.

## 2. Analysis of Human Lower Limb Gait Characteristics and Data Collection

The primary challenge in analyzing the gait characteristics of a lower limb exoskeleton is understanding the gait of the human body, as the exoskeleton must be highly compatible with human movement to effectively assist patients with lower limb disorders and improve rehabilitation outcomes.

### 2.1. Analysis of Human Lower Limb Gait Characteristics

**Figure 1:**
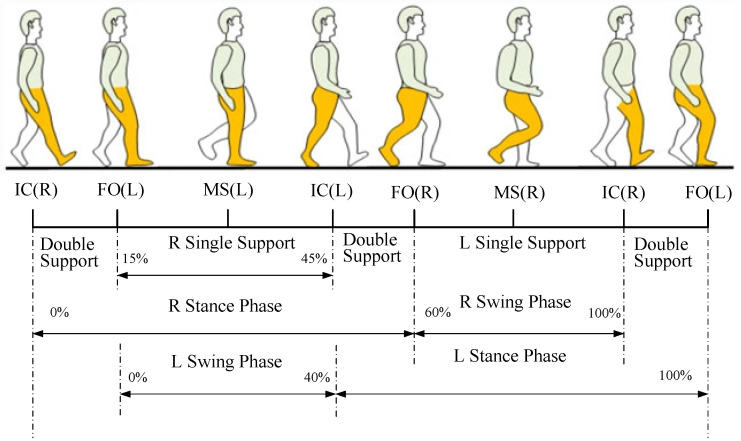
Schematic diagram of the human gait cycle (R: Right leg; L: Left leg; IC: Initial Contact; FO: Foot Off; MS: Mid-swing).

Walking is a routine activity for most individuals, but, for those with lower limb disabilities, it becomes an unattainable task. During walking, joint angle changes primarily occur in the sagittal plane. The process from one heel strike to the next constitutes a gait cycle [17] characterized by periodicity, coordination, and symmetry between the left and right legs. The biomechanical factors of natural gait involve the muscular strength and mechanical energy that control the body’s forward movement. These factors effectively absorb mechanical energy during foot strikes, reducing impact and controlling forward progression. As shown in Figure 1, the normal walking gait cycle consists of two phases: the stance phase and the swing phase, also referred to as the support and swing phases. The stance phase accounts for approximately 60% of the gait cycle, during which the foot is in contact with the ground, while the swing phase accounts for around 40%, during which the supporting leg lifts off the ground and swings forward.

During the entire support phase, single-leg support constitutes approximately 70%, while double-leg support accounts for around 30%. In the single-leg support phase, one foot remains in contact with the ground, bearing the body’s weight and propelling it forward. This phase begins when one foot strikes the ground and ends when the other foot strikes the ground. Muscle control of the foot, calf, and thigh is essential during this phase to support body weight and maintain balance. During the double-leg support phase, both feet are in contact with the ground, and the body’s weight is distributed between the two legs. This phase begins when the first foot strikes the ground and ends when the second foot lifts. During the double-leg support phase, the body’s center of gravity shifts forward, generating propulsive force that provides sufficient kinetic energy for the next phase, the swing phase. In individuals with lower limb movement disorders, the duration of the double-leg support phase is significantly prolonged to enhance stability during movement.

### 2.2. Data Collection of Joint Angles in the Human Lower Limb

Data collection of lower limb motion can provide detailed motion data, which serve as a solid foundation for gait analysis and the design of lower limb rehabilitation robots. The data collection is achieved through various sensors and technologies. Optical motion capture can be used, employing multiple high-speed cameras and markers (reflective spheres) to capture lower limb motion [18]. The 3D position of the markers is recorded by cameras, and joint angles, stride length, step width, and other parameters are calculated. Inertial motion capture can also be used, which involves placing Inertial Measurement Units (IMUs) such as accelerometers, gyroscopes, and magnetometers on different parts of the body to measure movement [19]. In addition, surface electromyographic (sEMG) signal collection is widely applied in gait analysis and rehabilitation robot design [20]. By using sEMG signals as control inputs, myoelectric control algorithms can be developed to enhance the responsiveness and naturalness of rehabilitation robots. The system used in this study is an optical 3D motion capture system. To ensure the accuracy and reliability of the collected data, we implemented a comprehensive calibration and validation protocol. The calibration process begins with daily system calibration using Optitrack’s standard calibration wand, which ensures precise camera positioning and optimal capture volume setup. For static validation, we measured known distances between fixed markers and verified marker position stability across all cameras. Dynamic validation was performed by comparing captured motion data with established biomechanical ranges and conducting real-time tracking verification during movement. To minimize random errors and improve data reliability, each measurement was repeated five times, and the results were averaged. Additionally, all collected data underwent cross-validation with existing literature data to ensure consistency with established gait patterns. This rigorous calibration and validation process ensures that our motion capture system provides accurate and reliable data for gait analysis and subsequent exoskeleton design.

#### 2.2.1. Experimental Platform

The experiments were conducted using an OptiTrack optical 3D motion capture system within a controlled indoor space measuring 5 m × 4.4 m × 2.6 m. The capture volume was monitored by six FLEX 3 infrared cameras positioned strategically around the space. The system provided 3D spatial positioning with a 16 m^2^ capture range, featuring a 56° horizontal and 46° vertical field of view. Each camera could track up to 80 points simultaneously, with the system capable of capturing up to 10 objects.

The cameras were mounted on a stable truss structure to ensure consistent positioning throughout data collection. Environmental controls included carpeted flooring and removal of reflective surfaces to minimize interference. Data collection was performed using Motive software Version2.0.0., which enabled real-time tracking and processing of 3D motion data with adjustable sampling frequencies. The system required marker points to be captured by at least two cameras simultaneously to ensure accurate spatial tracking.

#### 2.2.2. Experimental Procedure

To study the movement characteristics of the lower limbs and provide a theoretical basis for the structural design of lower limb exoskeleton rehabilitation robots, five subjects were selected to participate in a lower limb joint motion capture experiment (age 22 ± 3 years, weight 60 ± 20 kg, height 170 ± 10 cm, foot length 250 ± 15 mm). Walking was chosen as the basic movement pattern, with a level surface as the primary constraint. The subjects wore dark-colored pants and black sports shoes, walking with their feet naturally parallel.

**Figure 2:**
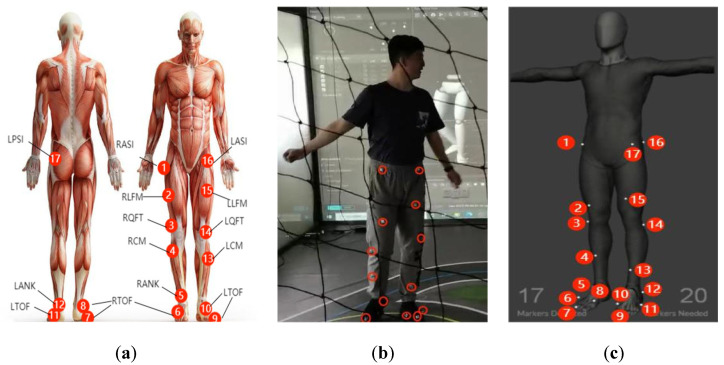
Experiment procedure: (**a**) distribution of muscle groups in human gait; (**b**) marker placement locations; (**c**) tracking of the moving target points.

The OptiTrack optical 3D motion capture system used in this study primarily captures the movement trajectory of reflective marker points in space. Therefore, reflective markers need to be attached to the subject during the capture process. Since the system captures the markers rather than the subject itself, the clothing worn by the subject is an important factor. If the subject wears loose clothing that shakes excessively during motion capture, it may obstruct the markers, which could affect the accuracy of the capture. Based on the muscle activity patterns during human walking [21], as shown in Figure 2a, the markers were placed on the body’s joints. The correct placement of markers is crucial for accurate motion capture. Markers should be positioned at specific, easily identifiable locations, with the precise marker placement parameters listed in Table 1.

A total of 17 markers were attached to the lower limbs in a non-linear, non-symmetrical arrangement, as shown in Figure 2b, to define the lower limb skeletal model and obtain 3D coordinates for motion data capture. Then, set the camera to raw grayscale mode, increase the exposure and LED settings, and adjust the focal length of the six FLEX 3 infrared cameras to stably track the moving target, as shown in Figure 2c.

**Figure 3:**
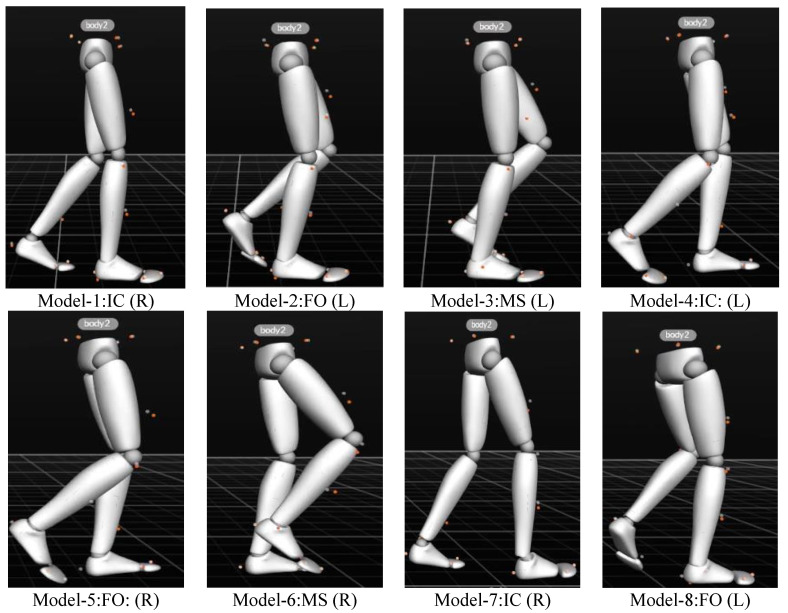
Gait model.

Using the Optitrack optical 3D motion capture system, the moment of heel strike is determined by checking whether the marker on the heel is near stationary. The gait cycle motion data for each subject are retained and extracted, as shown in the gait model in Figure 3. Figure 3 has a sampling frequency of 100 Hz and two complete gait cycles of 2.5 s but the time between each gait interval is not fixed and the gait interval is between 0.16 s and 0.18 s. The gait model of Figure 3 corresponds to the gait cycle of Figure 1. The data are then exported using the Motive software.

#### 2.2.3. Data Processing

During the data collection process, due to factors such as marker jitter, body movement, and environmental interference, the gait signal may contain significant high-frequency noise, such as spikes or sudden changes. This can cause difficulties in gait analysis, cycle segmentation, and feature extraction. Therefore, it is necessary to remove some of the data.

The design of the Butterworth low-pass filter is based on the principle of the Butterworth filter. It uses a specific frequency response curve that allows for the retention of the low-frequency components of the input signal while filtering out the high-frequency components, thus achieving noise reduction and signal smoothing [22]. To remove noise, a fourth-order Butterworth low-pass filter is used to process the trajectory of the reflective marker points. The Butterworth low-pass filter can be represented by the following formula for the square of the amplitude with respect to frequency:

(1) $${\left|H(w)\right|}^{2}=\frac{1}{1+{\left(\frac{w}{w_{c}}\right)}^{2n}}=\frac{1}{1+\varepsilon^{2}{\left(\frac{w}{w_{p}}\right)}^{2n}}$$

where *n* = the order of the filter;

*ω_c_* = cutoff frequency; 

*ω_p_* = edge frequency of the passband; 

$\frac{1}{1+\varepsilon^{2}}={\left|H(w)\right|}^{2}$ the value at the edge of the passband.

The frequency response curve of a Butterworth filter is maximally flat within the passband, without ripple, and gradually decreases to zero in the stopband. We set the filter order to 4 and the cutoff frequency to 5 Hz to eliminate high-frequency noise during the acquisition process. Since the duration of each gait cycle is not consistent, for the convenience of subsequent analysis, we use resampling to standardize the duration of all gait cycles to 2.5 s.

#### 2.2.4. Experimental Results and Analysis

Through the study of the basic structure of the lower limbs, it is known that, during normal walking, changes in joint angles primarily occur in the sagittal plane. Therefore, we focus on studying the joint angles within the sagittal plane. We exported the joint motion data obtained from Motive software, processed the trajectory of the reflective markers using a Butterworth filter, and performed curve fitting in Matlab to obtain the joint angle change equations for the left and right legs.

**Figure 4:**
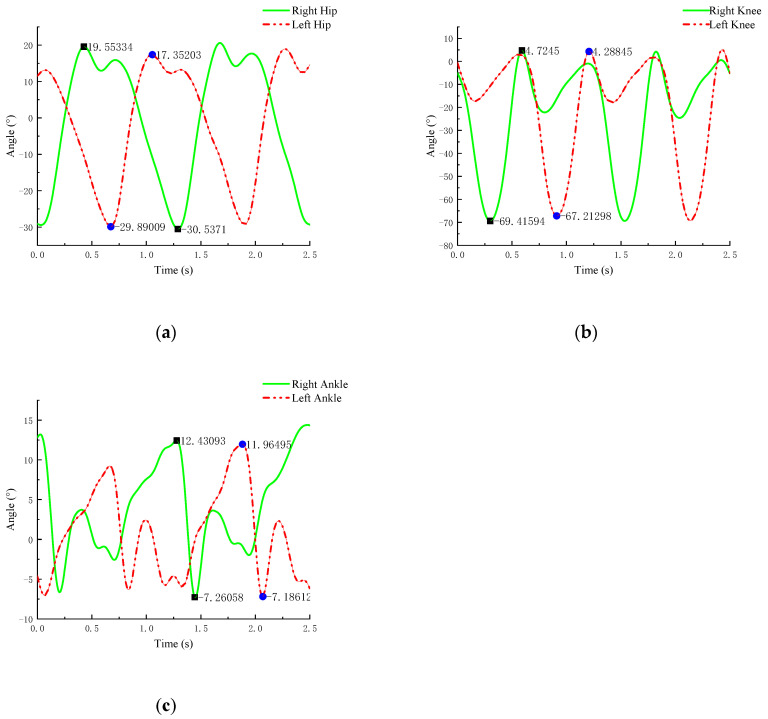
Joint angle change curves within the gait cycle: (**a**) hip joint angle change; (**b**) knee joint angle change; (**c**) ankle joint angle change.

At the same time, through the study of human gait cycles, it was found that the main factors influencing joint angle changes during walking are stride length and gait cycle. Due to factors such as height and weight, there are certain differences in joint angle changes among the five subjects, but the phase characteristics and waveform patterns of the joint angle change curves normalized by time were highly consistent in all subjects. Therefore, we selected individual data from a representative subject (height 170 cm, weight 65 kg, foot length 250 mm). The physical parameters of this subject were close to the mean values of the experimental group (height 170 ± 10 cm, weight 60 ± 20 kg), and the curve of the joint angle changes is shown in Figure 4.

From Figure 4, it can be observed that, although the joint angle changes in both legs during walking are not identical, they exhibit symmetry and coordination within the gait cycle. Each leg undergoes similar joint angle changes during its respective gait cycle. The following conclusions can be drawn from the analysis:

(1) Figure 4a shows the hip joint flexion and extension. In the initial stage of the gait cycle, the hip joint undergoes a significant flexion (bending) motion, which maximizes the energy storage of the stride and generates sufficient reactive force to propel the body forward. In the latter half of the walking cycle, the hip joint undergoes extension (upright motion), helping to convert the forward momentum of the body into upward momentum.

(2) Figure 4b shows the knee joint flexion and extension. In the initial stage of the gait cycle, the knee joint undergoes a certain degree of flexion, reducing the impact force between the lower limbs and the ground, which helps alleviate the burden on the knee. In the second phase, the knee joint undergoes a degree of extension (upright), which helps accelerate and stabilize the stride.

(3) Figure 4c shows the ankle joint dorsiflexion and plantarflexion. During the gait cycle, the ankle joint undergoes dorsiflexion (toe pointing upward) in the initial stage to gradually move the body’s center of gravity forward. In the latter half of the cycle, the ankle joint undergoes plantarflexion (toe pointing downward) to stabilize the stride and support the body’s weight. The range of joint angle changes for each joint is shown in Table 2.

The analysis results indicate that the conditions for maintaining a normal, natural walking gait are as follows: Throughout the entire gait cycle, the hip joint exhibits the following sequence of movements: initial flexion→maximum extension→flexion. The knee joint follows the movement pattern of a small initial flexion→maximum flexion, with full extension. The ankle joint exhibits the following sequence: initial small plantarflexion→large dorsiflexion→large plantarflexion→small dorsiflexion. By collecting normal human movement data and gait analysis, data support is provided for the design of the robot. The joint range of motion of a normal person serves as a reference for determining the joint activity range of the lower limb exoskeleton robot. By collecting these data, the maximum joint angle of the robot can be established to ensure that its range of motion does not exceed the natural limits of human movement, thus preventing damage to the patient’s joints or muscles. At the same time, the rehabilitation robot needs to adapt to the different rehabilitation needs of patients. The normal human movement data provide a “normal movement trajectory” for the robot, which can serve as a reference template during the rehabilitation process. Based on the patient’s rehabilitation progress, the robot can gradually adjust its movement patterns, either mimicking the normal movement trajectory or adjusting according to the patient’s capabilities.

## 3. Design of Lower Limb Exoskeleton Rehabilitation Robot

In the previous section, we obtained the joint motion angle curves of the human body through optical 3D motion capture experiments. These data provide important evidence for the structural design of the lower limb exoskeleton rehabilitation robot. Next, based on the preliminary data, we designed the overall structure of the lower limb exoskeleton rehabilitation robot.

### 3.1. Design Principles of the Lower Limb Exoskeleton Rehabilitation Robot

The primary target population for the lower limb exoskeleton rehabilitation robot includes individuals with lower limb disabilities and movement disorders. The robot serves the purpose of rehabilitation training and walking assistance. To ensure better integration of the exoskeleton with the human body, we have already obtained the angle variation ranges for the hip, knee, and ankle joints during normal walking through the motion capture experiments and motion data analysis in the previous chapter. To achieve optimal performance, the structural design of the exoskeleton rehabilitation robot should follow these principles:

(1) Bionic Principle: The robot’s configuration should resemble the human body structure. Its spatial dimensions should closely match those of the human body. To accommodate people of different heights and weights, the exoskeleton robot should be highly versatile, featuring adjustable mechanisms for the thigh and calf lengths.

(2) Safety and Reliability Principle: The exoskeleton robot primarily works in active or passive rehabilitation modes, driving the lower limbs to simulate normal movement trajectories and restore human mobility. To prevent secondary injuries to the patient, the structural strength of the lower limb exoskeleton rehabilitation robot must be safe and reliable.

(3) Compliance Principle: Walking is a non-patterned activity for the human body. During movement, forces should be applied as smoothly as possible to minimize large fluctuations. Therefore, the design of the lower limb exoskeleton rehabilitation robot should ensure compliance in terms of dynamics, providing a smooth and controlled motion experience.

Based on these principles, our design incorporates several innovative features that distinguish it from existing commercial products. These innovations specifically address key challenges in rehabilitation robotics while maintaining adherence to the fundamental design principles:

(1) Enhanced Joint Compliance Mechanism: Unlike traditional rigid joint designs, our exoskeleton implements a novel crank–slider mechanism that provides enhanced compliance, particularly in the knee and ankle joints. This design better mimics natural human joint movement and reduces impact forces during walking, directly supporting both the bionic and compliance principles.

(2) Optimized Weight Distribution System: We developed an innovative weight distribution system that strategically balances the exoskeleton’s mass across multiple support points. This system significantly reduces user fatigue during extended use and ensures more natural movement patterns, enhancing both safety and compliance aspects.

(3) Multi-Adaptive Adjustment System: Our design features a comprehensive adjustment mechanism that allows for precise customization to different body sizes. This includes independent length adjustments for thigh and calf segments, variable width adjustment at the hip level, and quick-release mechanisms for easy donning and doffing.

(4) Hybrid Actuation Integration: The exoskeleton integrates both passive and active elements to optimize energy efficiency while maintaining natural movement patterns. This includes spring-based energy storage in the ankle joint combined with motor-driven assistance at the hip and knee joints, providing a unique balance between power and efficiency.

(5) Comprehensive Safety Features: Building upon the safety principle, we incorporated multiple layers of protection, including mechanical joint limits, emergency stop mechanisms, and real-time monitoring systems.

### 3.2. Design Standards for the Dimensions of Lower Limb Exoskeleton Rehabilitation Robots

The exoskeleton robot needs to match the height of the human body, and its design should consider ergonomics and the comfort requirements of the wearer. According to the Ma’s torso-to-leg length index [23], although there are some differences in the reference data provided by different countries, the variation is not significant. Therefore, we refer to the Chinese standard GB/T1000-1998 “Human Body Dimensions for Adults” (as the wearers in this study are adults) [24]. Currently, there are significant challenges and high costs associated with the design and manufacturing of lower-limb exoskeleton robots, making it difficult to create customized models. When designing wearable lower-limb exoskeleton robots, it is important to fully consider the wearer’s height and body characteristics. The design should allow for adjustments in key dimensions within a certain range to accommodate different body types and meet the needs of various wearers. The proportional size information for different body parts in China is shown in Table 3.

As shown in the table above, the thigh length of males is concentrated between 428 and 505 mm, the lower leg length of males is concentrated between 338 and 403 mm, and the hip width of males is concentrated between 282 and 334 mm. For females, the thigh length is concentrated between 402 and 476 mm, the lower leg length is concentrated between 290 and 346 mm, and the hip width is concentrated between 290 and 346 mm.

### 3.3. Joint Design of Lower Limb Exoskeleton Rehabilitation Robot

The joint design of the exoskeleton robot needs to mimic the characteristics of the human joints as much as possible, while also ensuring the compactness and safety of the structure.

#### 3.3.1. Mechanism Degree of Freedom Solution

Human lower limb movement involves three major joints: the hip, knee, and ankle, each operating within a specific biomechanical range. Calculating the degrees of freedom ensures that the lower limb exoskeleton robot aligns with the natural motion of human joints at the kinematic level, preventing unnecessary movement restrictions or additional burdens on the wearer. This paper calculates the degrees of freedom of the mechanism using a modified Grübler–Kutzbach (G–K) equation:

(2) $$F=m(n-j-1)+\sum_{i=1}^{j}f_{i}-C_{r}$$

In the equation: *F* represents the degrees of freedom of the mechanism. *m* denotes the dimensionality of the motion space (*m* = 3 for planar mechanisms, *m* = 6 for spatial mechanisms). *n* refers to the number of links in the mechanism, including the frame. *j* indicates the number of kinematic pairs in the mechanism. *f_i_* represents the degrees of freedom of the *i*-th kinematic pair. *C_r_* denotes the number of redundant constraints.

According to studies in human anatomy and biomechanics, the human hip joint has three degrees of freedom. Following the principles of biomimetic design, the hip joint should ideally accommodate all three degrees of freedom. However, considering the target users of lower limb exoskeleton rehabilitation robots and the relatively small range of motion in adduction/abduction and internal/external rotation, these movements are omitted, retaining only the flexion/extension motion pair.

During normal walking, the rotational movement of the knee joint is minimal. Therefore, the knee joint can be considered a single-axis joint with only flexion/extension movement in the sagittal plane.

The structure of the ankle joint is similar to that of the hip joint, possessing three degrees of freedom: dorsiflexion/plantarflexion, internal/external rotation, and inversion/eversion. However, considering human movement characteristics, internal/external rotation and inversion/eversion have a minor impact on overall movement, so only the dorsiflexion/plantarflexion degree of freedom is retained in the design.

Thus, for this mechanism, *m* = 6, *n* = 7 (including one frame, two thighs, two lower legs, and two feet), and *j* = 6 (including the hip, knee, and ankle joints of both legs). Each joint has a degree of freedom of *f_i_* = 1. There are no redundant constraints, i.e., *C_r_* = 0. Substituting these values into the above equation, we obtain the following:

$$F=m(n-j-1)+\sum_{i=1}^{j}f_{i}-C_{r}=6\times (7-6-1)+6-0=6$$

Thus, the degrees of freedom of the bilateral lower limb exoskeleton robot are calculated to be 6, which is fewer than those of the human body. However, considering that the lower limb exoskeleton robot is primarily used for assistance and rehabilitation training, 6 degrees of freedom are sufficient.

#### 3.3.2. Hip Joint

**Figure 5:**
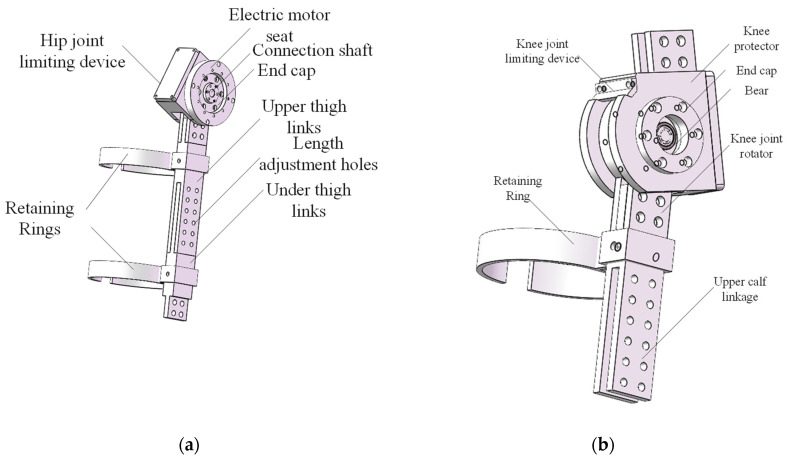

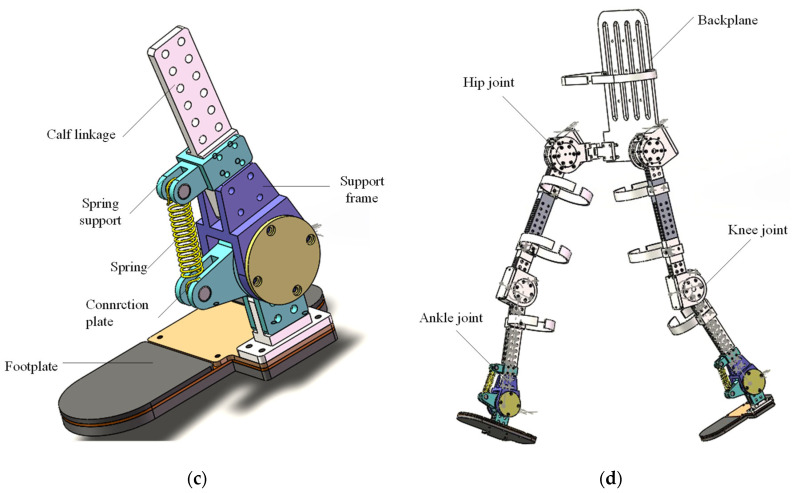
Lower limb exoskeleton rehabilitation robot joint design: (**a**) hip joint; (**b**) knee joint; (**c**) ankle joint; (**d**) overall 3D structure.

The structure of the hip joint is shown in Figure 5a. An adjustable waist belt is designed around the waist, which can be tailored to fit different human waist and hip sizes. Additionally, taking into account that most elderly individuals or patients may have some degree of lumbar issues, the waist device also serves to protect the lower back and prevent strain. Furthermore, a thigh fixation strap is installed on the joint linkage to securely fasten the thigh and lower limb structure with a flexible strap, improving both the reliability of the wear and the comfort of the wearer.

#### 3.3.3. Knee Joint

The structure of the knee joint is shown in Figure 5b. The knee joint is the most significant in terms of assistance and load-bearing in the human body and experiences considerable impact forces during the ground contact phase of walking, with the maximum impact force being more than four times the body weight. As such, the design of the knee joint is similar to that of the human patella, featuring a protective knee shell that can withstand large loads and impacts, while also incorporating angle limiters, to provide overload protection.

#### 3.3.4. Ankle Joint

The structure of the ankle joint is shown in Figure 5c. It consists of components connected to the lower leg and foot and uses a spring to generate an assistive torque during dorsiflexion. The design allows for adjustable initial angle and dorsiflexion assistive torque. During certain phases of gait, the spring can store energy and release it at an appropriate moment. The overall 3D structure of the lower limb exoskeleton rehabilitation robot is shown in Figure 5d.

### 3.4. Kinematic Analysis of Lower Limb Exoskeleton Rehabilitation Robot

The purpose of performing kinematic analysis on the lower limb exoskeleton rehabilitation robot is to ensure that the robot can accurately and effectively perform the required movement tasks, thereby achieving the desired rehabilitation functions. Through kinematic analysis, the laws of the time and spatial changes of the components of the lower limb exoskeleton robot can be clarified, providing a theoretical foundation for the design of the compliant drive system and control system of the robot [25]. Kinematic analysis can be divided into forward kinematics and inverse kinematics. Forward kinematics analysis calculates the position and orientation (pose) of the end-effector (i.e., the ankle joint) based on the given joint variables (such as joint angles) of the exoskeleton robot [26]. Inverse kinematics analysis, on the other hand, calculates the required joint variables based on a given target pose of the end-effector, which is crucial for achieving specific motion trajectories [27].

#### 3.4.1. D-H Kinematic Modeling

When performing a kinematic analysis of the lower limb exoskeleton rehabilitation robot, various methods can be used to study and calculate the robot’s motion characteristics. Common methods include the standard D-H parameter method [28], the motion chain method [29], the vector method [30], and the iterative method [31]. Currently, the most widely used multi-rigid-body kinematic description method is the D-H parameter method. Therefore, in this section, the D-H parameter method is used for the kinematic analysis of the lower limb exoskeleton rehabilitation robot.

**Figure 6:**
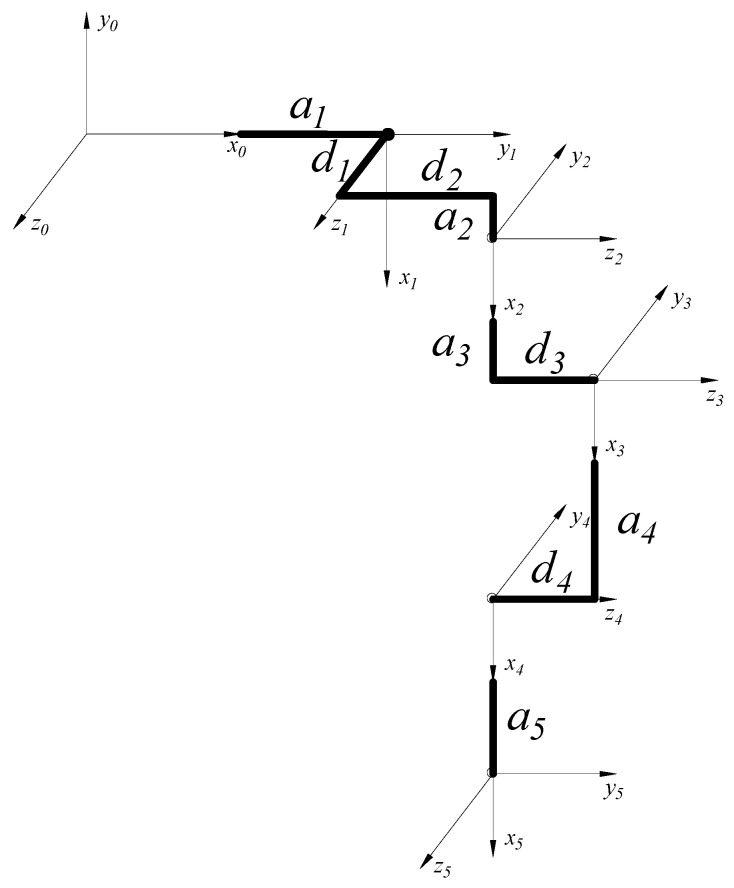
Schematic diagram of the kinematic coordinate system configuration for the left leg of the lower limb exoskeleton.

Based on the analysis of the basic structure of the human lower limb and gait cycle in Section 2, combined with the standard D-H parameter method, a schematic diagram of the lower limb exoskeleton kinematic coordinate system configuration is shown in Figure 6. Since the left and right legs of the human body are symmetrical, we will only analyze the left leg here.

The coordinate system selection for Figure 6 is as follows: The 0-coordinate system represents the basic coordinate system of the left leg. The 1-coordinate system represents the abduction/adduction degree of freedom at the left hip joint, where the *z*_1_ axis of the left leg is parallel and aligned with the *z*_0_ axis, and the *x*_1_ axis is the common perpendicular direction between *z*_0_ and *z*_1_. The 2-coordinate system represents the flexion/extension degree of freedom at the left hip joint, where the *z*_2_ axis of the left leg is perpendicular to the *z*_1_ axis and points to the right, and the *x*_2_ axis is the common perpendicular direction between *z*_1_ and *z*_2_. The 3-coordinate system represents the flexion/extension degree of freedom at the left knee joint, where the *z*_3_ axis of the left leg is parallel and aligned with the *z_2_* axis, and the *x*_3_ axis is the common perpendicular direction between *z_2_* and *z*_3_. The 4-coordinate system represents the dorsiflexion/plantarflexion degree of freedom at the left ankle joint, where the *z*_4_ axis of the left leg is parallel and aligned with the *z*_3_ axis, and the *x*_4_ axis is the common perpendicular direction between *z*_3_ and *z*_4_. The 5-coordinate system represents the eversion/inversion degree of freedom at the left ankle joint, where the *z*_5_ axis of the left leg is perpendicular to the *z*_4_ axis, and the *x*_5_ axis is the common perpendicular direction between *z*_4_ and *z*_5_. Based on the selected coordinate systems, the D-H parameter table for the left leg can be obtained, as shown in Table 4.

#### 3.4.2. Forward Kinematic Analysis and Simulation of the Lower Limb Exoskeleton Rehabilitation Robot

- Forward Kinematic Analysis

We substitute the D-H parameters from Table 4 into expression, thereby obtaining the transformation matrix for the left leg from the hip joint to the ankle joint.

(3) T50=T10T21T32T43T54=nxoxaxPxnyoyayPynzozazPz0001

where

$$\left\{\begin{array}{l} n_{x}=c\theta_{1}s\theta_{5}+s\theta_{1}c\theta_{5}c\left(\theta_{2}+\theta_{3}+\theta_{4}\right)\\ n_{y}=s\theta_{1}s\theta_{5}-c\theta_{1}c\theta_{5}c\left(\theta_{2}+\theta_{3}+\theta_{4}\right)\\ n_{z}=-s\left(\theta_{2}+\theta_{3}+\theta_{4}\right)c\theta_{5} \end{array}\right.\left\{\begin{array}{l} o_{x}=c\theta_{1}c\theta_{5}-s\theta_{1}s\theta_{5}c\left(\theta_{2}+\theta_{3}+\theta_{4}\right)\\ o_{y}=s\theta_{1}c\theta_{5}+s\theta_{5}c\theta_{1}c\left(\theta_{2}+\theta_{3}+\theta_{4}\right)\\ o_{z}=s\left(\theta_{2}+\theta_{3}+\theta_{4}\right)s\theta_{5} \end{array}\right.\left\{\begin{array}{l} a_{x}=s\theta_{1}s\left(\theta_{2}+\theta_{3}+\theta_{4}\right)\\ a_{y}=-c\theta_{1}s\left(\theta_{2}+\theta_{3}+\theta_{4}\right)\\ a_{z}=c\left(\theta_{2}+\theta_{3}+\theta_{4}\right) \end{array}\right.$$

$$\left\{\begin{array}{l} P_{x}=a_{1}+\left(d_{2}+d_{3}+d_{4}\right)c\theta_{1}+a_{2}s\theta_{1}+a_{3}c\theta_{2}s\theta_{1}+a_{4}s\theta_{1}c\left(\theta_{2}+\theta_{3}\right)+a_{5}s\theta_{1}c\left(\theta_{2}+\theta_{3}+\theta_{4}\right)\\ P_{y}=\left(d_{2}+d_{3}+d_{4}\right)s\theta_{1}-a_{2}c\theta_{1}-a_{3}c\theta_{2}c\theta_{1}-a_{4}c\theta_{1}c\left(\theta_{2}+\theta_{3}\right)-a_{5}c\theta_{1}c\left(\theta_{2}+\theta_{3}+\theta_{4}\right)\\ P_{z}=d_{1}-a_{3}s\theta_{2}-a_{4}s\left(\theta_{2}+\theta_{3}\right)-a_{5}s\left(\theta_{2}+\theta_{3}+\theta_{4}\right) \end{array}\right.$$

In the equation, *Px*, *Py*, and *Pz* represent the positions of the end effector of the lower limb exoskeleton’s left leg along the *x*, *y*, and *z* axes, respectively.

- Based on MATLAB Robotics forward kinematics simulation

The MATLAB Robotics Toolbox is a comprehensive set of functions for robot modeling, simulation, control, and analysis [32]. It provides a complete environment for the design and development of robotic systems, supporting everything from simple motion planning to the implementation of complex robot control algorithms. In the research process, we developed our own SolidWorks model and planned to load it into Simulink for further simulation. However, for initial verification, we chose to use the standard manipulator model from the MATLAB Robotics Toolbox for simulation, in order to quickly validate the kinematic feasibility. Since the models in this toolbox have been widely used and validated, selecting it as a comparison model in the early stages effectively reduces potential model errors.

Based on the adult human body dimensions, the D-H parameter table for the left leg in Table 4 is derived, and the joint-link parameters are selected as follows:

*a*_1_ = 0.167 m, *a_2_* = 0.050 m, *a*_3_ = 0.505 m, *a*_4_ = 0.403 m, *a*_5_ = 0.028 m

*d*_1_ = 0.078 m, *d_2_* = 0.004 m, *d*_3_ = 0.014 m, *d*_4_ = 0.013 m

**Figure 7:**
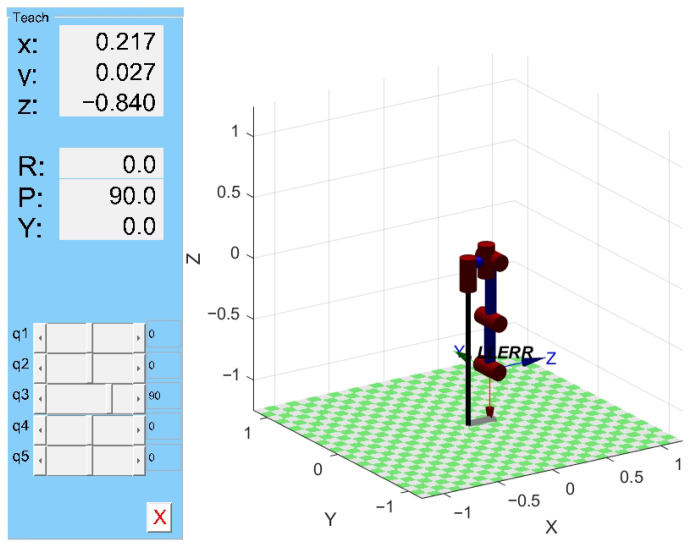
Three-dimensional model of the left leg.

First, let us define the D-H parameters. Each link’s D-H parameters include ‘d’: Link offset (along the *z*-axis of the previous link); ‘a’: Link length (along the *x*-axis of the current link); ‘alpha’: Link twist angle (about the *x*-axis of the current link); ‘theta’: Joint angle (this is a variable and is not set in the Link definition). Next, using the ‘SerialLink’ function, we input the list of link parameters to create the robot’s serial chain model. Finally, by using ‘robot.fkine()’, we calculate the end-effector pose for the given joint angles. This allows us to obtain the 3D model of the left leg, as shown in Figure 7.

By comparing with the link model established in Figure 6, it can be seen that the model is correct. On the left side of the model, there is a teaching adjuster for the joint variables, which allows for manual adjustment of the motion range of each joint variable to observe the end joint position at different postures. We set the joint angles of the left leg as *θ* = [π/6, π/4, π/3, π/6, π/4]ᵀ. By substituting these values into MATLAB, we can obtain the end-effector position and orientation as follows:

(4) T50=−0.183−0.96590.1830.1943−0.6830.25830.6830.3731−0.70710.8327−0.7071−0.76820001

From this, it can be seen that the end-effector position is as follows:

(5) $$\left[\begin{array}{l} p_{x}\\ p_{y}\\ p_{z} \end{array}\right]=\left[\begin{array}{c} 0.1943\\ 0.3731\\ -0.7682 \end{array}\right]$$

By substituting *θ* = [π/6, π/4, π/3, π/6, π/4]ᵀ into Equation (6), and performing the calculation, we can also obtain the following:

(6) T50=T10T21T32T43T54=−0.183−0.96590.1830.1943−0.6830.25830.6830.3731−0.70710.8327−0.7071−0.76820001

Through simulation and calculation, it can be observed that the results are consistent. Therefore, this further verifies that the kinematic model of the left leg of the lower limb exoskeleton is correct.

- Velocity and Acceleration Calculation

The Jacobian matrix is a core concept in multivariable calculus, used to describe the partial derivatives of a vector-valued function with respect to multiple independent variables. It has widespread applications in optimization, robotics, control systems, physical modeling, and other fields. In the previous analysis, we obtained the end joint position. Next, we use the Jacobian matrix to calculate the velocity and acceleration of the end joint.

The Jacobian matrix is defined as follows:

(7) $$J(\theta )=\left[\begin{array}{lllll} \frac{\partial x}{\partial \theta_{1}} & \frac{\partial x}{\partial \theta_{2}} & \frac{\partial x}{\partial \theta_{3}} & \frac{\partial x}{\partial \theta_{4}} & \frac{\partial x}{\partial \theta_{5}}\\ \frac{\partial y}{\partial \theta_{1}} & \frac{\partial y}{\partial \theta_{2}} & \frac{\partial y}{\partial \theta_{3}} & \frac{\partial y}{\partial \theta_{4}} & \frac{\partial y}{\partial \theta_{5}}\\ \frac{\partial z}{\partial \theta_{1}} & \frac{\partial z}{\partial \theta_{2}} & \frac{\partial z}{\partial \theta_{3}} & \frac{\partial z}{\partial \theta_{4}} & \frac{\partial z}{\partial \theta_{5}} \end{array}\right]$$

Based on the previously derived end joint position, substituting it into the above equation, we compute the partial derivatives of each component with respect to the variable *θ*. After calculation, we obtain the following:

$$J(\theta )=\left[\begin{array}{lllll} -0.12 & 0.45 & 0.38 & 0.22 & 0.15\\ 0.25 & -0.32 & 0.40 & 0.35 & 0.20\\ 0.10 & 0.18 & 0.22 & 0.30 & 0.12 \end{array}\right]$$

Calculate the end joint velocity as follows:

(8) $$\;X\;˙=J(\theta )\cdot \dot{\theta }$$

In the equation, *θ* represents the joint angular velocity, which refers to the instantaneous velocity of rotation for each joint in robotic kinematic analysis. Given the joint angles *θ*, we can roughly estimate the joint’s angular velocity and angular acceleration.

The joint angular velocity is as follows:

(9) $$\dot{\theta }={\left[0.1,0.2,0.15,0.1,0.05\right]}^{T}rad/s$$

Thus, we can obtain the following:

$$\dot{X}=\left[\begin{array}{lllll} -0.12 & 0.45 & 0.38 & 0.22 & 0.15\\ 0.25 & -0.32 & 0.40 & 0.35 & 0.20\\ 0.10 & 0.18 & 0.22 & 0.30 & 0.12 \end{array}\right]\left[\begin{array}{l} 0.1\\ 0.2\\ 0.15\\ 0.1\\ 0.05 \end{array}\right]=\left[\begin{array}{c} 0.58\\ 0.0465\\ 0.041 \end{array}\right]$$

Therefore, the following is true:

$$\dot{x}=0.58m/s\;\;\;\;\dot{y}=0.0465m/s\;\;\;\;\dot{z}=0.041m/s$$

Calculate the end joint acceleration as follows:

(10) $$\ddot{X}=J(\theta )\cdot \ddot{\theta }+\dot{J}(\theta )\cdot \dot{\theta }$$

The joint angular acceleration is as follows:

(11) $$\ddot{\theta }={\left[0.01,0.02,0.015,0.01,0.005\right]}^{T}rad/s^{2}$$

Thus, we can obtain the following:

$$\dot{X}=\left[\begin{array}{lllll} -0.12 & 0.45 & 0.38 & 0.22 & 0.15\\ 0.25 & -0.32 & 0.40 & 0.35 & 0.20\\ 0.10 & 0.18 & 0.22 & 0.30 & 0.12 \end{array}\right]\left[\begin{array}{l} 0.01\\ 0.02\\ 0.015\\ 0.01\\ 0.005 \end{array}\right]+\left[\begin{array}{lllll} 0.002 & 0.005 & 0.004 & 0.002 & 0.001\\ 0.003 & 0.002 & 0.006 & 0.003 & 0.002\\ 0.001 & 0.004 & 0.005 & 0.006 & 0.002 \end{array}\right]=\left[\begin{array}{c} 0.1320\\ 0.0148\\ 0.0113 \end{array}\right]$$

Therefore, the following is true:

$$\ddot{x}=0.1320m/s^{2}\;\;\;\;\ddot{y}=0.0148m/s^{2}\;\;\;\;\ddot{z}=0.0113m/s^{2}$$

From the above calculations, it can be seen that the robot’s velocity in the *x*-direction, i.e., the robot’s forward speed, is slower than the normal walking speed. The speed in the *y*-direction is slightly higher, which may cause instability. At the same time, because the speed is relatively slow, the acceleration is smaller. However, considering the robot’s primary application for rehabilitation training of patients or elderly individuals, a slower walking speed is preferable and meets the usage requirements.

#### 3.4.3. Inverse Kinematic Analysis and Simulation of the Lower Limb Exoskeleton Rehabilitation Robot

- Inverse Kinematic Analysis

In practical robotic applications, the end-effector’s desired position is usually known, and we need to control the robot to reach that position. Therefore, inverse kinematics analysis is of great significance. There are various methods to solve for the joint variables of each link based on the end-effector’s position, including algebraic methods, analytical methods, numerical methods, or optimization algorithms (such as genetic algorithms, particle swarm optimization, etc.). In this study, we use the algebraic method to solve for the joint angles of the lower limb exoskeleton.

- Solve for the joint angle *θ*_1_

Multiply both sides of Equation (3) by T−110θ1, thus obtaining the following:

(12) T−110θ1T50=T21θ2T32θ3T43θ4T54θ4

After the transformation, we obtain the following:

(13) $$\begin{array}{l} \left[\begin{array}{llll} s\theta_{1}n_{x}-c\theta_{1}n_{y} & s\theta_{1}o_{x}-c0_{1}o_{y} & s\theta_{1}a_{x}-c\theta_{1}a_{y} & s\theta_{1}P_{x}-c\theta_{1}P_{y}-a_{1}s\theta_{1}\\ c\theta_{1}n_{x}+s\theta_{1}n_{y} & c\theta_{1}o_{x}+s\theta_{1}o_{y} & c\theta_{1}a_{x}+s\theta_{1}a_{y} & c\theta_{1}P_{x}+s\theta_{1}P_{y}-a_{1}c\theta_{1}\\ n_{z} & o_{z} & a_{z} & P_{z}-d_{1}\\ 0 & 0 & 0 & 1 \end{array}\right]=\\ \left[\begin{array}{llll} c\left(\theta_{2}+\theta_{3}+\theta_{4}\right)c\theta_{5} & -c\left(\theta_{2}+\theta_{3}+\theta_{4}\right)s\theta_{5} & s\left(\theta_{2}+\theta_{3}+\theta_{4}\right) & a_{2}+a_{3}c\theta_{2}+a_{4}c\left(\theta_{2}+\theta_{3}\right)+a_{5}c\left(\theta_{2}+\theta_{3}+\theta_{4}\right)\\ s\theta_{5} & c\theta_{5} & 0 & d_{2}+d_{3}+d_{4}\\ -s\left(\theta_{2}+\theta_{3}+\theta_{4}\right)c\theta_{5} & s\left(\theta_{2}+\theta_{3}+\theta_{4}\right)s\theta_{5} & c\left(\theta_{2}+\theta_{3}+\theta_{4}\right) & -a_{3}s\theta_{2}-a_{4}s\left(\theta_{2}+\theta_{3}\right)-a_{5}s\left(\theta_{2}+\theta_{3}+\theta_{4}\right)\\ 0 & 0 & 0 & 1 \end{array}\right] \end{array}$$

By comparing the element in the second row and fourth column of the two matrices, we can obtain the following:

(14) $$c\theta_{1}\left(P_{x}-a_{1}\right)+s\theta_{1}P_{y}=d_{2}+d_{3}+d_{4}$$

Through calculation, the value of the joint angle *θ*_1_ can be obtained as follows:

(15) $$\theta_{1}=\arctan2\left(d_{2}+d_{3}+d_{4},\pm \sqrt{{\left(P_{x}-a_{1}\right)}^{2}+{P_{y}}^{2}-{\left(d_{2}+d_{3}+d_{4}\right)}^{2}}\right)-\arctan2\left(P_{x}-a_{1},P_{y}\right)$$

- Solve for the joint angle *θ*_3_

In Equation (6), by comparing the elements in the first row fourth column, second row fourth column, and third row fourth column, we can obtain the following:

(16) $$\left\{\begin{array}{l} s\theta_{1}\left(P_{x}-a_{1}\right)-c\theta_{1}P_{y}=a_{2}+a_{3}c\theta_{2}+a_{4}c\left(\theta_{2}+\theta_{3}\right)+a_{5}c\left(\theta_{2}+\theta_{3}+\theta_{4}\right)\\ P_{z}-d_{1}=-a_{3}s\theta_{2}-a_{4}s(\theta_{2}+\theta_{3})-a_{5}s\left(\theta_{2}+\theta_{3}+\theta_{4}\right)\\ c\theta_{1}\left(P_{x}-a_{1}\right)+s\theta_{1}P_{y}=d_{2}+d_{3}+d_{4} \end{array}\right.$$

Through calculation, the value of the joint angle *θ*_3_ can be obtained as follows:

(17) $$\theta_{3}=\arccos\frac{\left\{\begin{array}{l} {\left(P_{x}-a_{1}\right)}^{2}+{P_{y}}^{2}+{\left(P_{z}-d_{1}\right)}^{2}-2a_{1}s\theta_{1}\left(P_{x}-a_{1}\right)+2a_{2}c\theta_{1}P_{y}\\ +a_{2}^{2}-a_{3}^{2}-a_{4}^{2}-{\left(d_{2}+d_{3}+d_{4}\right)}^{2} \end{array}\right\}}{2a_{3}a_{4}}$$

- Solve for the joint angle *θ_2_*

Multiply both sides of Equation (3) by T−130θ1+θ2+θ3 on the left, thus obtaining the following:

(18) T−130θ1+θ2+θ3T50=T43θ4T54θ4

By following the method used to solve for the joint angle *θ*_1_, we can obtain the following:

(19) $$\theta_{2}=\arctan2\left(r,\pm \sqrt{{r_{1}}^{2}+{r_{2}}^{2}-r^{2}}\right)-\arctan2\left(r_{1},r_{2}\right)$$

where

$$\left\{\begin{array}{l} r_{1}=2a_{3}\left(s\theta_{1}\left(P_{x}-a_{1}\right)-P_{y}c\theta_{1}-a_{3}\right)\\ r_{2}=2a_{3}\left(P_{z}-d_{1}\right)\\ r={\left(s\theta_{1}\left(P_{x}-a_{1}\right)+a_{2}\right)}^{2}+c\theta_{1}{P_{y}}^{2}+{\left(P_{z}-d_{1}\right)}^{2}+{a_{3}}^{2}-{a_{4}}^{2}-2s\theta_{1}\left(P_{x}-a_{1}\right)c\theta_{1}P_{y}+2a_{2}c\theta_{1}P_{y} \end{array}\right.$$

- Solve for the joint angle *θ*_4_, *θ*_5_

We assume that, during walking, the body component connecting the left and right hip joints of the lower limb exoskeleton rehabilitation robot always remains horizontal, and the lower limb exoskeleton always stays vertically upright. Meanwhile, when the foot of the lower limb exoskeleton rehabilitation robot is in contact with the ground, it remains in full-sole contact. Therefore, we can obtain the following:

(20) $$\left\{\begin{array}{c} \theta_{2}+\theta_{3}+\theta_{4}=\frac{\pi }{2}\\ \theta_{1}+\theta_{5}=0 \end{array}\right.$$

Based on the above analysis, we can derive the inverse kinematics equation for the left leg of the lower limb exoskeleton.

(21) $$\left\{\begin{array}{l} \theta_{1}=\arctan2\left(d_{2}+d_{3}+d_{4},\pm \sqrt{{\left(P_{x}-a_{1}\right)}^{2}+{P_{y}}^{2}-{\left(d_{2}+d_{3}+d_{4}\right)}^{2}}\right)-\arctan2\left(P_{x}-a_{1},P_{y}\right)\\ \begin{array}{l} \theta_{2}=\arctan2\left(r,\pm \sqrt{{r_{1}}^{2}+{r_{2}}^{2}-r^{2}}\right)-\arctan2\left(r_{1},r_{2}\right)\\ \left\{\begin{array}{l} r_{1}=2a_{3}\left(s\theta_{1}\left(P_{x}-a_{1}\right)-P_{y}c\theta_{1}-a_{3}\right)\\ r_{2}=2a_{3}\left(P_{z}-d_{1}\right)\\ r={\left(s\theta_{1}\left(P_{x}-a_{1}\right)+a_{2}\right)}^{2}+c\theta_{1}{P_{y}}^{2}+{\left(P_{z}-d_{1}\right)}^{2}+{a_{3}}^{2}-{a_{4}}^{2}-2s\theta_{1}\left(P_{x}-a_{1}\right)c\theta_{1}P_{y}+2a_{2}c\theta_{1}P_{y} \end{array}\right. \end{array}\\ \theta_{3}=\arccos\frac{\left\{\begin{array}{l} {\left(P_{x}-a_{1}\right)}^{2}+{P_{y}}^{2}+{\left(P_{z}-d_{1}\right)}^{2}-2a_{1}s\theta_{1}\left(P_{x}-a_{1}\right)+2a_{2}c\theta_{1}P_{y}\\ +a_{2}^{2}-a_{3}^{2}-a_{4}^{2}-{\left(d_{2}+d_{3}+d_{4}\right)}^{2} \end{array}\right\}}{2a_{3}a_{4}}\\ \theta_{4}=\frac{\pi }{2}-\theta_{2}-\theta_{3}\\ \theta_{5}=-\theta_{1} \end{array}\right.$$

- Based on MATLAB Robotics inverse kinematics simulation

Using the forward kinematics simulation method, we also employ the Robotics Toolbox to verify whether the above analysis is correct.

First, define the D-H parameters and choose the same values as in the forward kinematics. The D-H parameters for each link are consistent with those mentioned earlier. After creating the robot model, define the end-effector’s target pose using ’T_goal’. The target homogeneous transformation matrix is generated using the ’transl()’ and ’trotz()’ functions. The joint angles for the given target pose are then calculated using the ’ikine()’ function. Since inverse kinematics may have multiple solutions, by default, ’ikine()’ returns one of the possible solutions.

**Figure 8:**
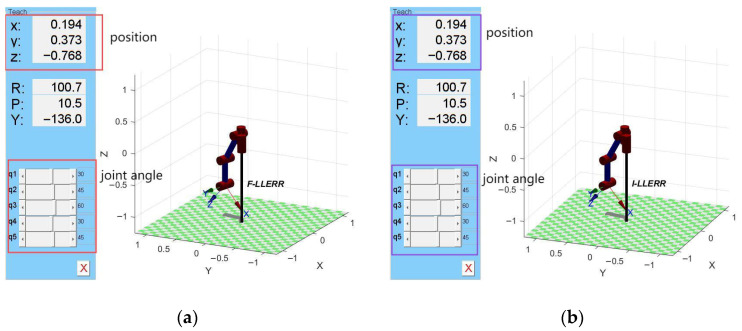
Inverse kinematics verification: (**a**) forward kinematics model; (**b**) inverse kinematics model.

In the forward kinematics verification, we calculated the end-effector positions *Px* = 0.1943, *Py* = 0.3731, and *Pz* = −0.7682 by giving the joint angles *θ* = [π/6, π/4, π/3, π/6, π/4]ᵀ. The corresponding 3D model of the forward kinematics for the left leg is shown in Figure 8a. Now, using the above position as the target position, we solve for the joint angles, and the obtained joint angles (in radians) are as follows:

$$\theta =\left[\begin{array}{ccccc} 0.5236 & 0.7854 & 1.0472 & 0.5236 & 0.7854 \end{array}\right]$$

The conversion formula between radians and degrees is as follows:

(22) $$\deg rees=radians\times \frac{180}{\pi }$$

Thus, the calculated joint angles are essentially consistent with the given joint angles of the left leg. The resulting 3D model of the inverse kinematics for the left leg is shown in Figure 8b.

By comparing Figure 8a,b, it can be seen that the input and output of the end-effector of the left leg in both the forward kinematics and inverse kinematics simulations are perfectly coincident, indicating that the inverse kinematics equations are correct.

### 3.5. PID Control Strategy Based on BP Fuzzy Neural Network

During the assistive walking process of a lower limb exoskeleton rehabilitation robot, the environment is not constant. If the PID values remain unchanged, it may not provide effective feedback. The BP neural network can learn and adapt to the dynamic changes of the system, autonomously adjusting the control parameters to ensure that the control system maintains good performance under different working conditions. By combining fuzzy control with neural networks, the system can effectively handle highly nonlinear and complex uncertainties, thereby improving control accuracy and robustness. BP fuzzy neural network PID control has strong anti-interference capabilities, effectively suppressing external disturbances and noise in the system, and maintaining system stability.

#### 3.5.1. Fuzzy PID Controller’s Domain and Membership Functions

**Figure 9:**
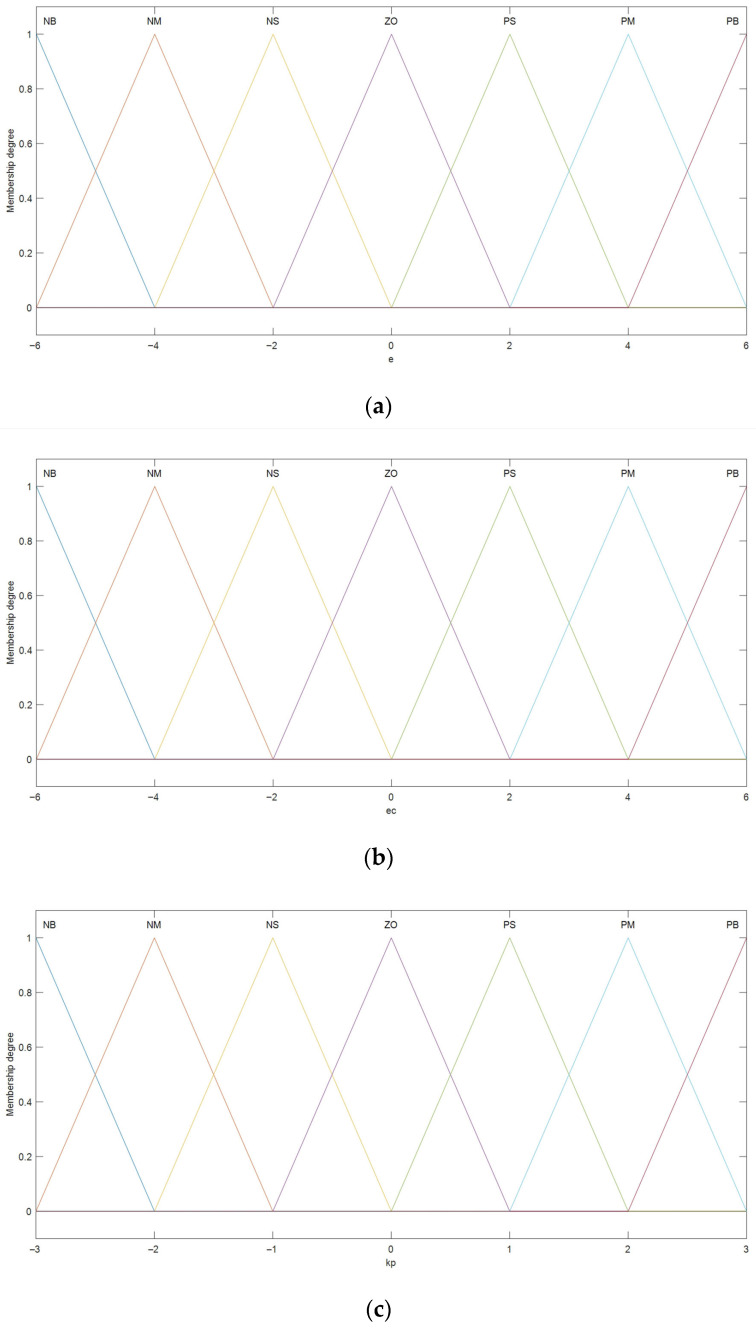

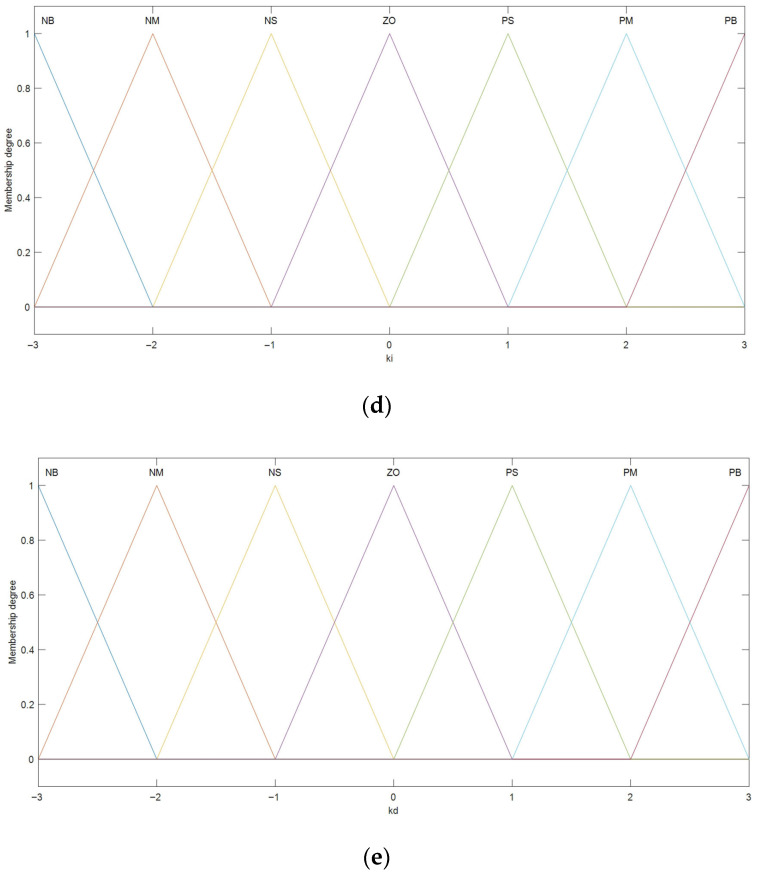
Membership function plots: (**a**) membership function of input variable *e*; (**b**) membership function of input variable *ec*; (**c**) membership function of output variable *k_p_*; (**d**) membership function of output variable *k_i_*; (**e**) membership function of output variable *k_d._*

The inputs to the fuzzy PID controller are the error (*e*) and the rate of change of error (*ec*), where the error is obtained by comparing the feedback value and the control setpoint. The outputs are the three tuning parameters of the PID controller: kp, ki, and kd. The domains for the fuzzy reasoning of *e* and *ec* are both [−6, 6], corresponding to linguistic values {Negative Big (NB), Negative Medium (NM), Negative Small (NS), Zero (ZO), Positive Small (PS), Positive Medium (PM), Positive Big (PB)}. The membership functions used are triangular (trimf). The domains for the fuzzy reasoning of kp, ki, and kd are [−3, 3], with corresponding linguistic values {NB, NM, NS, ZO, PS, PM, PB}. The membership function curves are shown in Figure 9.

#### 3.5.2. BP Fuzzy Neural Network PID Controller Design

**Figure 10:**
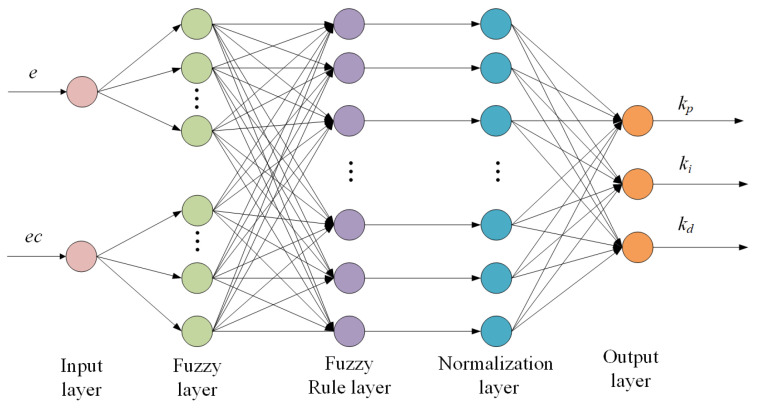
Structure of BP neural network.

Fuzzy PID control has strong adaptability, but its autonomous learning ability is poor. The advantage of the fuzzy neural network PID controller is that it can automatically learn fuzzy rules and control strategies through the neural network, without the need to manually design fuzzy rules. This makes it more suitable for complex systems that require adaptive learning. In the previous section, we fuzzified the three parameters of the PID controller *k_p_*, *k_i_*, and *k_d_*, and used fuzzy rule inference to adjust these parameters in real-time, enabling the controller to adapt to the system’s non-linearity and dynamic changes. The BP neural network is used to adjust the fuzzy controller’s membership functions and fuzzy rule base online, allowing the system to self-adapt and optimize control performance. The network structure diagram of the BP neural network PID controller is shown in Figure 10, consisting of the input layer, fuzzification layer, fuzzy rule layer, normalization layer, and output layer.

The first layer is the input layer, which has two neurons representing the *e* and the *ec*. Here, *x*_1_ = *e*, *x*_2_ = *ec*. These two input signals are passed to the next layer, and the expression is as follows:

(23) $$\left\{\begin{array}{l} I_{j}=x_{j}\\ O_{j}=I_{j} \end{array}\right.$$

In the expression, *j* = 1, 2, *I_j_* represents the input of the *j*-th neuron node in the first layer, and *O_j_* represents the output of the *j*-th neuron node in the first layer.

The second layer is the fuzzification layer, consisting of seven neurons connected to (*e*) and (*ec*). This layer performs fuzzification to process the input signals before passing them to the next layer. The expression is as follows:

(24) $$\left\{\begin{array}{l} I_{k}=O_{j}\\ O_{k}=f_{jk}\left(x_{j}\right)=\exp\left[-\frac{{\left(O_{j}-m_{jk}\right)}^{2}}{2\theta_{jk}^{2}}\right] \end{array}\right.$$

In the expression, *k* = 1, 2, 3, ⋯, 7; *m_jk_* and *θ_jk_* represent the mean difference and standard deviation of the *k*-th membership function of the *j*-th input signal, respectively, and *I_k_* and O*_k_* represent the input and output of the second layer.

The third layer is the fuzzy rule layer, where each neuron represents a fuzzy rule between the input and output variables. Each neuron computes the rule’s fitness *A_l_*, and there are a total of 49 rules, expressed as follows:

(25) $$\left\{\begin{array}{l} I_{l}=O_{k}\\ O_{l}=A_{l}=\frac{1}{2}\sum_{j=1}^{2}f_{jk}\left(x_{j}\right) \end{array}\right.$$

In the expression, *l* = 1, 2, 3, ⋯, 49; *I_l_* and *O_l_* represent the input and output of the third layer, respectively.

The fourth layer is the normalization layer, consisting of 49 neurons. This layer normalizes the outputs of the 49 neurons from the third layer. The normalization is performed as follows:

(26) $$\begin{array}{l} I_{m}=O_{l}\\ O_{m}=\beta_{li}=A_{l}gf_{li}\left(z_{j}\right)\\ f_{li}\left(z_{j}\right)=\exp\left[-\frac{{\left(z_{j}-\mu_{li}\right)}^{2}}{2\sigma_{li}^{2}}\right] \end{array}$$

In the expression, *i* = 1, 2, 3, *m* = 1, 2, 3, ⋯, 49, *μ_li_* and *σ_li_* represent the mean difference and standard deviation of the membership function of the *i*-th output variable of the *l*-th rule, respectively, and *I_m_* and *O_m_* represent the input and output of the fourth layer.

The fifth layer is the output layer, which consists of three neurons, each representing an output variable. The expression is as follows:

(27) $$\left\{\begin{array}{l} I_{i}=O_{m}\\ O_{i}=Z_{i}=\frac{\int_{z_{i}}\beta_{li}z_{i}dz_{i}}{\int_{z_{i}}\beta_{li}dz_{i}}=\frac{\sum_{i=1}^{49}\sigma_{li}\mu_{li}A_{l}}{\sum_{i=1}^{49}\sigma_{li}A_{l}} \end{array}\right.$$

In the expression, *I_i_* and *O_i_* represent the input and output of the fourth layer, respectively.

#### 3.5.3. The Calculation of Joint Input and Output Torques

The dynamic model of the lower limb exoskeleton joints is represented using the Lagrange equation:

(28) $$\tau =M(\theta )\ddot{\theta }+C(\theta ,\dot{\theta })\dot{\theta }+G(\theta )+F(\dot{\theta })$$

where

τ is the joint input torque (generated by the actuator);

M(θ) is the inertia matrix;

$C(\theta ,\dot{\theta })$ is the Coriolis and centrifugal force matrix;

G(θ) is the gravitational term;

$F(\dot{\theta })$ is the friction term;

$\theta ,\dot{\theta },\ddot{\theta }$ represent joint angle, angular velocity, and angular acceleration, respectively.

The controller input is the trajectory tracking error (*e*) and the error rate of change (*ec*) $ec=\dot{e}$, with the output being the dynamically adjusted PID parameters *k_p_*, *k_i_*, *k_d_*. The input torque equation is as follows:

(29) $$\tau_{\mathrm{input}}=k_{p}\cdot e+k_{i}\cdot \int edt+k_{d}\cdot \frac{de}{dt}$$

**Figure 11:**
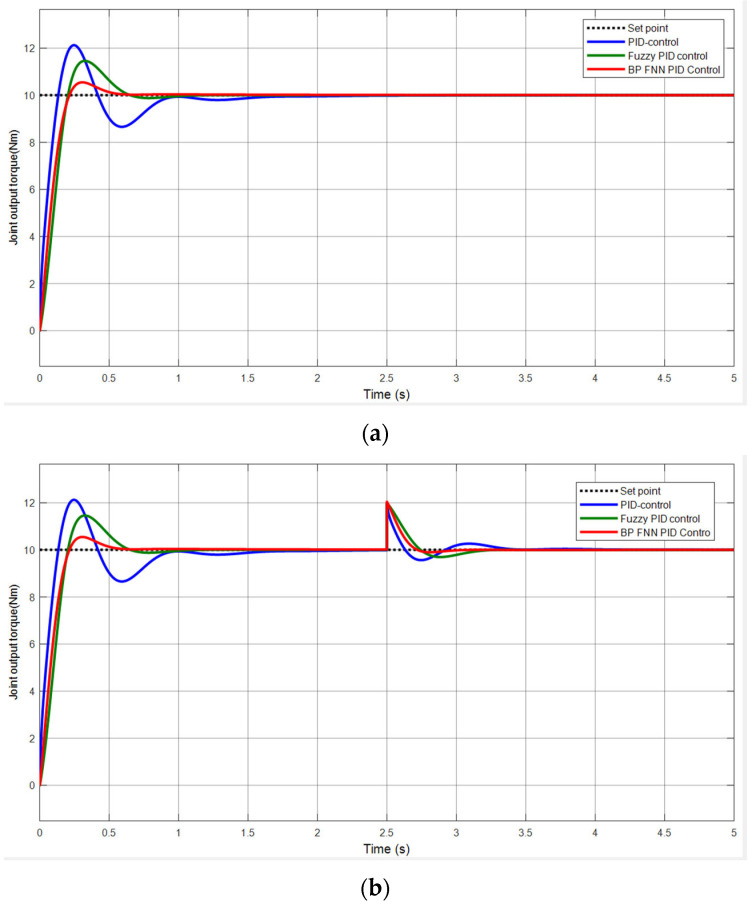
Simulink simulation results: (**a**) no disturbance; (**b**) with disturbance.

By combining the dynamic model and control equations, the output torque $\tau_{\mathrm{output}}$ must satisfy the following:

(30) $$\tau_{\mathrm{input}}=\tau_{\mathrm{output}}+\Delta \tau_{\mathrm{error}}$$

where $\Delta \tau_{\mathrm{error}}$ represents the impact of system disturbances or unmodeled dynamics. In the ideal case with no disturbances (Figure 11a), the following is true:

(31) $$\tau_{\mathrm{input}}=M(\theta )\ddot{\theta }+C(\theta ,\dot{\theta })\dot{\theta }+G(\theta )+F(\dot{\theta })$$

When external disturbances are present (Figure 11b), a disturbance compensation term must be introduced:

(32) $$\tau_{\mathrm{input}}=M(\theta )\ddot{\theta }+C(\theta ,\dot{\theta })\dot{\theta }+G(\theta )+F(\dot{\theta })+\tau_{\mathrm{disturbance}}$$

In the Simulink simulation model, the input torque is generated by the controller, and the output torque is calculated through the dynamic equations. The input torque (controller output) is as follows:

(33) $$\tau_{\mathrm{input}}=f_{\mathrm{BP}-\mathrm{FNN}-\mathrm{PID}}(e,ec)$$

where $f_{\mathrm{BP}-\mathrm{FNN}-\mathrm{PID}}$ is the nonlinear mapping function of the PID parameters by the fuzzy neural network.

The output torque (dynamic response) is as follows:

(34) $$\tau_{\mathrm{ouput}}=M(\theta )\ddot{\theta }+C(\theta ,\dot{\theta })\dot{\theta }+G(\theta )+F(\dot{\theta })$$

Next, we modeled a system simulation using MATLAB/Simulink based on the derived equations.

#### 3.5.4. BP Fuzzy Neural Network PID Control Simulation Analysis

A system simulation model is built using MATLAB/Simulink, which mainly consists of a fuzzy PID controller, disturbance signals, the controlled system (plant), and control setpoints. Simulink block diagrams are constructed to compare traditional PID control, fuzzy PID control, and BP fuzzy neural network PID control. After debugging, the following parameters are selected: fuzzification factors *k_e_* = 0.8 and *k_ec_* = 0.2; defuzzification factors *k_p_*_1_= 0.5, *k_i_*_1_= 8, and *k_d_*_1_= −0.1; initial PID values *k_p_*= 8, *k_i_* = 15, and *k_d_* = 0.5.

When a 10 Nm step signal is applied, the simulation time is set to 5 s, the output torque of the joint shown in Figure 11a represents the simulation without disturbances, and Figure 11b represents the simulation with disturbances.

From the simulation curve in Figure 11a, it can be observed that, when using traditional PID control, there is noticeable oscillation, with an overshoot of 21.2% and a settling time of approximately 0.96 s. When using fuzzy PID control, the oscillation is significantly reduced, with an overshoot of 14.6% and a settling time of 0.66 s. In contrast, when using BP fuzzy neural network PID control, there is no obvious oscillation, with an overshoot of only 5.5% and a settling time of 0.49 s. A comparison table of the simulation results for the three control methods is shown in Table 5.

In the actual walking process of the human body, various external factors can cause disturbances. To verify the system’s robustness, a disturbance is introduced at 2.5 s during system operation, and Figure 11b shows the simulation results. From the figure, it can be observed that, when a disturbance occurs, the BP fuzzy neural network PID controller responds the fastest with the shortest adjustment time, followed by the fuzzy PID controller, and the traditional PID controller has the slowest response. Under BP fuzzy neural network control, the oscillation amplitude is the smallest, while, under fuzzy PID control, the oscillation amplitude is similar to that of the BP fuzzy neural network PID. The oscillation amplitude is the largest under traditional PID control.

## 4. Wearable Testing of the Lower Limb Exoskeleton Rehabilitation Robot Prototype

**Figure 12:**
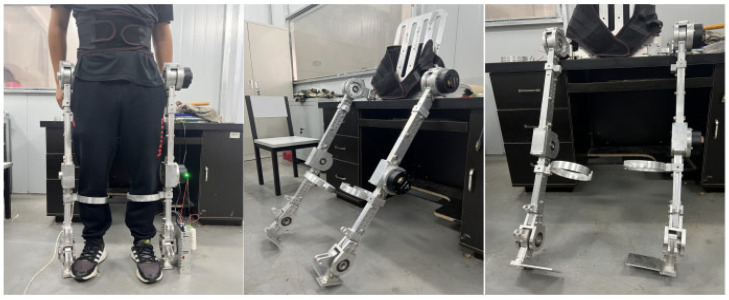
Prototype donning demonstration.

Based on the 3D model of the lower limb exoskeleton rehabilitation robot designed in the previous chapters, 7075 aluminum was chosen as the main material. The robot uses RMD-X8pro1:9V3 servo motors and CAN communication for control. The prototype of the lower limb exoskeleton rehabilitation robot, as shown in Figure 12, has been fabricated and assembled. The overall weight of the prototype is 25 kg, distributed as follows: lower limb segments (15 kg), actuators (7 kg), and control system (3 kg). The exoskeleton is designed for users with a height of 178 ± 5 cm and a weight of 79 ± 10 kg, with adjustable segments to accommodate height variations of ±10 cm. After simple training consisting of three 15 min sessions, the user can complete the donning process in 50 ± 5 s, making it relatively convenient compared to similar devices which typically require 2–3 min for donning.

**Figure 13:**
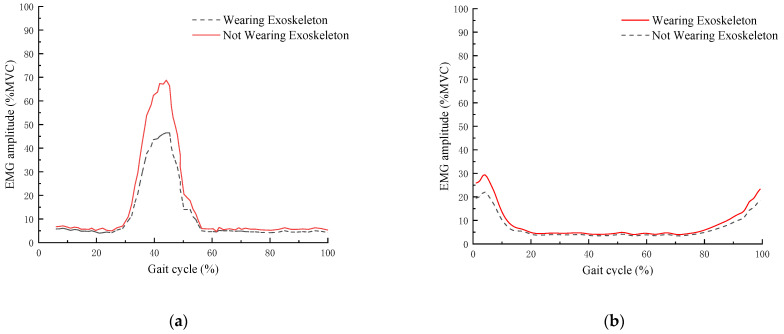

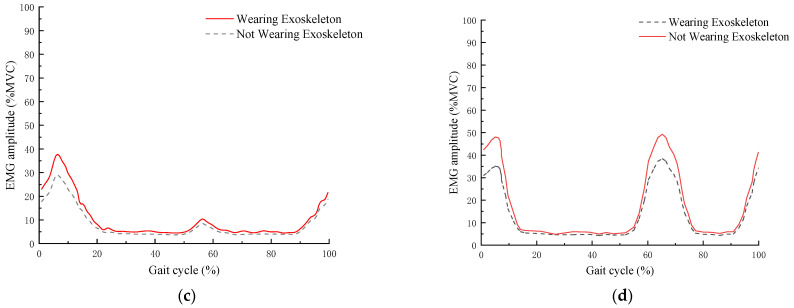
EMG amplitude (%MVC): (**a**) gastrocnemius; (**b**) biceps femoris; (**c**) rectus femoris; (**d**) tibialis anterior.

To validate the feasibility of the lower limb exoskeleton rehabilitation robot design, we conducted a comprehensive experimental evaluation on 15 healthy participants (age: 25 ± 3 years, height: 175 ± 5 cm, weight: 70 ± 10 kg). In the gait experiment, we collected electromyographic (EMG) activity (EMG amplitude) of major lower limb muscles under two conditions: wearing and not wearing the exoskeleton. Before the experiment, each participant performed a maximal voluntary contraction (MVC) test, during which the maximum EMG amplitude of the target muscles (gastrocnemius, biceps femoris, rectus femoris, and tibialis anterior) was recorded. The EMG data obtained during the experiment were normalized to MVC, and the final results were presented as a percentage of MVC (%MVC), as shown in Figure 13.

As can be seen from Figure 13, during normal walking, regardless of whether the exoskeleton is worn, the maximum EMG amplitude (%MVC) of the tibialis anterior occurs at the early stance phase; the maximum EMG amplitude (%MVC) of the gastrocnemius occurs during the mid-to-late stance phase; the maximum EMG amplitude (%MVC) of the rectus femoris occurs at the early stance phase; the maximum EMG amplitude (%MVC) of the biceps femoris occurs at the early stance phase. The maximum EMG amplitudes (%MVC) of these four muscles, in descending order, are as follows: gastrocnemius, tibialis anterior, rectus femoris, and biceps femoris. The average EMG amplitudes (%MVC) for both wearing exoskeleton and not wearing exoskeleton conditions are shown in Table 6.

The EMG data show that the activity of the gastrocnemius decreased by approximately 25%, the activity of the biceps femoris decreased by approximately 30%, the activity of the rectus femoris decreased by approximately 18%, and the activity of the tibialis anterior decreased by approximately 22%. These data indicate that the exoskeleton can effectively provide support to the lower limb muscles during the stance and push-off phases, reducing the muscle load. This suggests that the exoskeleton, by providing appropriate mechanical support, helps alleviate the user’s muscle burden, improves gait, and offers better rehabilitation outcomes.

**Figure 14:**
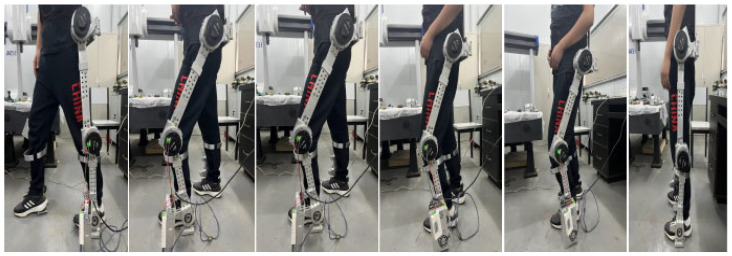
Walking gait test.

The testing process is shown in Figure 14, where subjects performed multiple walking trials including level ground walking (10 trials × 10 m), stair climbing (5 trials × 1 flight), and turning maneuvers (10 trials × 90°turns). After repeated gait tests, quantitative analysis showed the robot demonstrated a safe and stable walking process with a dynamic stability margin of 0.15 ± 0.02 m. However, compared to normal walking (1.2 ± 0.1 m/s), the speed was slower, with a walking speed of approximately 0.18 ± 0.02 m/s and a stride length of about 32 ± 3 cm. A complete gait cycle took around 1.8 ± 0.1 s. The step symmetry index reached 92 ± 3%, though the double support time increased by 45 ± 5% compared to normal walking (from 0.2 s to 0.29 ± 0.02 s). User feedback through standardized questionnaires (*n* = 15) indicated positive ratings for ease of donning/doffing (7.5 ± 0.5/10), perceived assistance level (7.2 ± 0.4/10), and comfort during use (6.8 ± 0.6/10), suggesting good potential for rehabilitation applications despite the current limitations in walking speed and weight. Energy consumption analysis showed an average power requirement of 150 ± 20 W during steady-state walking, with peak power demands of 250 ± 30 W during stair climbing.

## 5. Discussion

The lower limb exoskeleton rehabilitation robot developed in this study demonstrates both promising capabilities and areas for improvement when compared with existing systems. The key findings and their implications are discussed below. The kinematic design of our system, based on detailed gait analysis data from optical motion capture, shows good biomechanical compatibility with natural human movement. Compared to the LOKOMAT system [33], which is limited to treadmill-based training, our design enables overground walking, offering more practical rehabilitation scenarios. However, the current walking speed (0.18 m/s) is slower than that of commercial systems like HAL (0.3–0.4 m/s) [34], indicating room for improvement in actuation efficiency.

The EMG analysis results showed a 25–30% reduction in major muscle activation during walking, suggesting effective assistance comparable to the findings reported for the BLEEX system [35]. This validates our mechanical design approach and control strategy. However, the system’s weight (25 kg) remains a concern, being heavier than newer systems like the CR-35IA [36], which weighs approximately 20 kg. A significant innovation in our design is the integration of compliant elements in the ankle joint structure, similar to the approach used in StarIETH [37]. This feature contributes to the high step symmetry index (92 ± 3%) observed in our tests, suggesting good potential for maintaining natural gait patterns during rehabilitation. The positive user feedback scores (>7/10 for ease of use) also indicate successful implementation of human-centered design principles. Several limitations of the current study should be acknowledged:

(1) The testing was conducted only with healthy subjects, and clinical trials with actual rehabilitation patients are needed to validate therapeutic effectiveness.

(2) The limited walking speed may restrict the system’s applicability for patients at different stages of recovery.

(3) The relatively high weight of the system could pose challenges for long-duration use in clinical settings.

(4) The current design focuses primarily on sagittal plane motion, while human gait involves complex three-dimensional movements.

These findings have important implications for the future development of rehabilitation robotics. The success in achieving stable gait patterns suggests that our human–machine integration approach is viable, while the limitations identified provide clear directions for future improvements.

## 6. Conclusions

This paper presents the design of a lower limb exoskeleton rehabilitation robot for patients with lower limb disabilities, hemiplegia, or paraplegia. The design process begins by analyzing human gait characteristics, using the Quanser Optitrack optical 3D motion capture system to collect lower limb joint motion data. Based on these data, a virtual prototype is created with SolidWorks, followed by motion simulation analysis. A controller based on BP fuzzy neural network PID control is designed to ensure the stability of human walking. When using BP fuzzy neural network PID control, there is no significant oscillation, with an overshoot of 5.5% and a settling time of 0.49 s. Real-world wear tests verify the robot’s structural and functional reliability. The results show that, while the robot’s walking process is safe and stable, its speed (0.18 m/s) and stride length (32 cm) are slower and shorter than normal walking, with a gait cycle duration of 1.8 s. The robot also faces limitations such as a large size and weight (25 kg). Future work will focus on addressing these limitations through key improvements in three areas: mechanical optimization by replacing aluminum alloy components with carbon fiber composites to reduce weight by 40%, redesigning joint mechanisms to reduce degrees of freedom from 5 to 4, and adopting a modular design for easier customization; control system enhancement by developing an adaptive impedance control algorithm to increase walking speed by 50%, integrating real-time sEMG feedback, and introducing a learning-based controller to optimize gait patterns; and advanced gait planning by developing predictive algorithms, implementing smooth trajectory generation, and adding real-time obstacle avoidance. These improvements aim to achieve a walking speed of 0.5 m/s, reduce weight to 15 kg, and decrease double support time to 20% of normal walking.

## Figures and Tables

**Table 1:** Marker point placement.

Number	Marker Label	Definition	Position
1/16	RASI/LASI	Right/Left Anterior Auperior Iliac	Front right waist/front left waist
2/15	RLFM/LLFM	Right/Left Lateral Femoris Muscle	Outer side of the front thigh
3/14	RQFT/LQFT	Right/Left Quadriceps Femoris Tendon	Front side of the thigh muscles
4/13	RCM/LCM	Right/Left Calf Muscle	Back side of the calf
5	RANK	Right Lateral Ankle	Bone prominence on the outer side of the right ankle
6/7/8	RTOE	Right Toe	Tip of the right toe
9/10/11	LTOE	Left Toe	Tip of the left toe
12	LANK	Left Lateral Ankle	Bone prominence on the outer side of the left ankle
17	LPSI	Left Posterior Spine Iliac	Front outer side of the thigh muscles

**Table 2:** The range of motion of the joints in the lower limb.

Lower Limb	Joint	Range of Motion
Right leg	Hip joint	−31~20°
	Knee joint	−70~5°
	Ankle joint	−8~13°
Left leg	Hip joint	−30~18°
	Knee joint	−68~5°
	Ankle joint	−8~12°

**Table 3:** The lower limb dimensions of adult males and adult females in China (unit: mm).

Percentage	Male (18–60 Years Old)				Female (18–60 Years Old)			
	Height	Thigh Length	Lower Leg Length	Hip Width	Height	Thigh Length	Lower Leg Length	Hip Width
1	1543	413	324	273	1449	387	300	275
5	1583	428	338	282	1484	402	313	290
10	1604	436	344	288	1503	410	319	296
50	1678	465	369	306	1570	438	344	317
90	1754	496	396	327	1640	467	370	340
95	1775	505	403	334	1659	476	376	346
99	1814	523	419	346	1697	494	390	360

**Table 4:** D-H parameter table for the left leg.

*i*	*a_i_*	*α_i_*	*d_i_*	*θ_i_*
1	*a*_1_	0	*d*_1_	*θ*_1_ − *π*/2
2	*a*_2_	−*π*/2	*d*_2_	*θ*_2_
3	*a*_3_	0	*d*_3_	*θ*_3_
4	*a*_4_	0	*d*_4_	*θ*_4_
5	*a*_5_	*π*/2	0	*θ*_5_

**Table 5:** Comparison of simulation results.

Control Method	Overshoot	Settling Time
PID	21.2%	0.96 s
Fuzzy PID	14.6%	0.66 s
BP Neural Network PID	5.5%	0.49 s

**Table 6:** Comparison of average muscle EMG amplitude (%MVC) during walking with and without exoskeleton wear.

Muscle Name	Exoskeleton Wear Condition	Average EMG Amplitude (%MVC)
Gastrocnemius	No	65.5
	Yes	52.4
Biceps femoris	No	27.3
	Yes	19.1
Rectus femoris	No	34.2
	Yes	28.1
Tibialis anterior	No	46.7
	Yes	36.4

## Data Availability

Data are contained within the article.
